# Performance of the phonatory deviation diagram in the evaluation of rough and breathy synthesized voices^☆^^☆☆^

**DOI:** 10.1016/j.bjorl.2017.05.012

**Published:** 2017-07-05

**Authors:** Leonardo Wanderley Lopes, Jonas Almeida de Freitas, Anna Alice Almeida, Priscila Oliveira Costa Silva, Giorvan Ânderson dos Santos Alves

**Affiliations:** a Universidade Federal da Paraíba (UFPB), Departamento de Fonoaudiologia, João Pessoa, PB, Brazil; b Universidade Federal da Paraíba (UFPB), Curso de Fonoaudiologia, João Pessoa, PB, Brazil

**Keywords:** Voice quality, Acoustics, Phonation, Dysphonia, Voice disorders, Qualidade da voz, Acústica, Fonação, Disfonia, Distúrbios da voz

## Abstract

**Introduction:**

Voice disorders alter the sound signal in several ways, combining several types of vocal emission disturbances and noise. The phonatory deviation diagram is a two-dimensional chart that allows the evaluation of the vocal signal based on the combination of periodicity (jitter, shimmer, and correlation coefficient) and noise (Glottal to Noise Excitation) measurements. The use of synthesized signals, where one has a greater control and knowledge of the production conditions, may allow a better understanding of the physiological and acoustic mechanisms underlying the vocal emission and its main perceptual-auditory correlates regarding the intensity of the deviation and types of vocal quality.

**Objective:**

To analyze the performance of the phonatory deviation diagram in the discrimination of the presence and degree of roughness and breathiness in synthesized voices.

**Methods:**

871 synthesized vocal signals were used corresponding to the vowel /ɛ/. The perceptual-auditory analysis of the degree of roughness and breathiness of the synthesized signals was performed using visual analogue scale. Subsequently, the signals were categorized regarding the presence/absence of these parameters based on the visual analogue scale cutoff values. Acoustic analysis was performed by assessing the distribution of vocal signals according to the phonatory deviation diagram area, quadrant, shape, and density. The equality of proportions and the chi-square tests were performed to compare the variables.

**Results:**

Rough and breathy vocal signals were located predominantly outside the normal range and in the lower right quadrant of the phonatory deviation diagram. Voices with higher degrees of roughness and breathiness were located outside the area of normality in the lower right quadrant and had concentrated density.

**Conclusion:**

The normality area and the phonatory deviation diagram quadrant can discriminate healthy voices from rough and breathy ones. Voices with higher degrees of roughness and breathiness are proportionally located outside the area of normality, in the lower right quadrant and with concentrated density.

## Resumo

**Introdução:**

Os distúrbios de voz alteram o sinal sonoro de diversas formas, combinando variados tipos de perturbações e ruído na emissão vocal. O diagrama de desvio fonatório é um gráfico bidimensional que possibilita a avaliação do sinal vocal a partir da combinação de medidas de periodicidade (*jitter, shimmer* e coeficiente de correlação) e de ruído (*Glottal to Noise Excitation*). A utilização de sinais sintetizados, onde se tem um maior controle e conhecimento das condições de produção, pode possibilitar uma maior compreensão dos mecanismos fisiológicos e acústicos subjacentes à emissão vocal e seus principais correlatos perceptivo-auditivos quanto à intensidade do desvio e tipos de qualidade vocal.

**Objetivo:**

Analisar o desempenho do diagrama de desvio fonatório na discriminação da presença e do grau de rugosidade e soprosidade em vozes sintetizadas.

**Método:**

Foram utilizados 871 sinais vocais sintetizados correspondentes à vogal/ɛ/. Realizou-se a análise perceptivo-auditiva do grau de rugosidade e soprosidade dos sinais sintetizados, com uma escala visual analógica. Posteriormente, os sinais foram categorizados quanto à presença/ausência desses parâmetros a partir dos valores de corte da escala visual analógica. A análise acústica foi realizada por meio da avaliação da distribuição dos sinais vocais de acordo com a área, quadrante, forma e densidade do diagrama de desvio fonatório. Executou-se o teste de igualdade de proporções e o teste Qui-quadrado para comparar as variáveis.

**Resultados:**

Sinais vocais rugosos e soprosos localizaram-se predominantemente fora da área de normalidade e no quadrante inferior direito do diagrama de desvio fonatório. Vozes com maiores graus de rugosidade e soprosidade localizaram-se fora da área de normalidade, no quadrante inferior direito e apresentaram densidade concentrada.

**Conclusão:**

A área de normalidade e o quadrante do diagrama de desvio fonatório são capazes de discriminar vozes saudáveis de rugosas e soprosas. Vozes com maior grau de rugosidade e soprosidade localizam-se proporcionalmente fora da área de normalidade, no quadrante inferior-direito e com densidade concentrada.

## Introduction

Traditionally, vocal assessment includes the investigation and integration of perceptual-auditory, laryngeal, aerodynamic, acoustic, and self-assessment data.^1^, ^2^ Specifically, perceptual-auditory evaluation and acoustic analysis are the main tools used by the speech therapist/audiologist to characterize the vocal quality deviation observed in voice disorders.^3^

Studies in the area of voice disorder evaluation and diagnosis aim to investigate three essential clinical issues^3^: the ability of the measure to determine the presence/absence of a voice disorder (diagnosis); the evidence that the test used can determine the origin (etiology) of a voice disorder; and the ability of a measure to determine the extent (intensity) of a voice disorder.

The perceptual-auditory voice assessment includes from the definition of the present deviation intensity to the emission and predominant vocal quality, in case of deviated emissions. The descriptors “roughness”, “breathiness” and “tension” are universally used^4^, ^5^ to characterize dysphonic voices, showing a correlation in the physiological and acoustic planes.^6^, ^7^, ^8^ However, the roughness and breathiness parameters are considered more robust, whereas tension is a less reliable quality with great inter-rater variability, which justifies its omission in some perceptual-auditory evaluation protocols.^9^, ^10^

The acoustic analysis corresponds to the sound signal recording, which is the complex product of the non-linear interaction of the biomechanical and aerodynamic properties of the vocal production system.^8^ It provides an indirect estimate of the vibratory patterns of the vocal folds, the vocal tract, and its different adjustments, contributing to the task of vocal quality analysis and classification.^11^, ^12^, ^13^, ^14^

Jitter and shimmer are among the main acoustic measures based on linear models of vocal production and used in the clinical context.^15^ These are measures that analyze the fundamental frequency disturbance index, that is, the control of vocal fold vibrations (jitter), and the amplitude disturbance index, which is related to glottic resistance (shimmer).^16^, ^17^

In addition to disturbance measures, noise measurements such as Glottal to Noise Excitation (GNE) and Harmonic-Noise Ratio (HNR) are also widely used in the clinical context,^8^, ^18^, ^19^ as they demonstrate whether the vocal signal originates from vocal fold vibrations or the presented air current (GNE), as well as of the regular signal of the vocal folds in relation to the irregular signal of the vocal folds and the vocal tract, correlating the harmonic noise versus the wave noise component (HNR).^17^, ^19^, ^20^

In general, a deviant emission tends to combine different components of noise and disturbance, so that studies using combined measures may better represent the auditorily perceived vocal quality deviation.^8^, ^16^, ^20^, ^21^, ^22^, ^23^

In this context, the Phonatory Deviation Diagram (PDD) or hoarseness diagram (in its original version)^24^, ^25^, ^26^ offers the possibility of the combined analysis of disturbance measurements (jitter, shimmer, and correlation) and noise (GNE), making it an important tool for the evaluation and monitoring of voice disorders.^17^, ^27^, ^28^, ^29^, ^30^

One of the great challenges of vocal assessment is the integrated analysis of data, which includes the acoustic and perceptual-auditory information.^31^ One of the possible solutions suggested for a better understanding of the associations between the acoustic and perceptual phenomena related to the vocal signal is the development of researches with voices generated by synthesizers.^32^

Synthesized voices have highly controlled and known acoustic properties and production conditions, which contributes to the understanding of the mechanisms underlying the auditorily perceived vocal quality deviation. Synthesizers simulate vocal production deviations such as roughness, breathiness, and tension, from the manipulation of disturbance parameters, noise, and tension/symmetry differences between the vocal folds, respectively.^33^

Therefore, considering that the identification of the presence and degree of roughness and breathiness are part of the clinical vocal evaluation routine, that PDD is an important tool in the evaluation and monitoring of voice disorders, and that the use of synthesized signals allows greater control of the stimulus and can elucidate conditions underlying the perceived deviation, the aim of this research is to analyze the performance of PDD in the discrimination of the presence and degree of roughness and breathiness in synthesized voices.

For this purpose, two hypotheses were raised: (1) there are differences in the PDD parameters regarding the identification of voices with and without roughness and breathiness; (2) there are differences in the PDD parameters regarding the identification of signals with different degrees of roughness and breathiness.

## Methods

### Study design

This is a documented descriptive, and cross-sectional study carried out at the Voice Laboratory of the Speech Therapy and Audiology Department of a university. It was evaluated and approved by the Research Ethics Committee of the institution, under Opinion n. 508200/2013.

### Sample

This study used a set of synthesized voices developed by the VoiceSim synthesizer.^33^ The synthesizer consists of a computer system containing a vocal fold model and a representation of the vocal tract in the format of concatenated tubes, through which an acoustic wave propagates.^32^

Vocal deviations of roughness and breathiness were produced from the manipulation of acoustic parameters of fundamental frequency disturbance (flutter, tremor, and wow), additive noise and tension asymmetry between the vocal folds.^33^

Roughness was generated by manipulating the duration of the cycle of glottic excitation and jitter, with the introduction of a stochastic disturbance in the vocal fold tissue tension, using the formula: Δ*K* = *αεK*; where /*α*/ is a scale parameter, /*ɛ*/ is a random variable, and /*K*/ is a coefficient of vocal fold stiffness.

Breathiness was generated with the insertion of additive noise, according to the formula: Δ*μ* = *bεμ* where /*μ*/ is the glottal airflow rate, /*b*/ is a scale parameter, and /*ɛ*/ is a random variable, similar to jitter.

The tension asymmetry parameters between the vocal folds, subglottic pressure and vocal fold separation were also controlled during the production of these synthesized signals. For more details on the synthesizer, please refer to the available literature.^33^

The speech material of the synthesized stimuli was the vowel /ɛ/ sustained for 3 s. This vowel was chosen because it is commonly used in vocal and laryngeal evaluation procedures in Brazil,^34^ also considering that it is an oral, medium, open, and unrounded vowel, considered the most medium vowel of Brazilian Portuguese,^34^ which allows a more neutral and intermediate position of the vocal tract.

Therefore, 871 synthesized vocal signals were used, of which 426 (48.8%) were female and 446 (51.2%) were male signals, with different combinations of the previously mentioned acoustic parameters.

### Procedures

The acoustic analysis was performed using the VoxMetria software, version 4.5 h, by CTS Informática (Pato Branco, Paraná, Brazil), in the vocal quality module. The PDD was used for this evaluation, in order to analyze the distribution of vocal signals according to area, quadrant, shape, and density.

**Figure 1:**
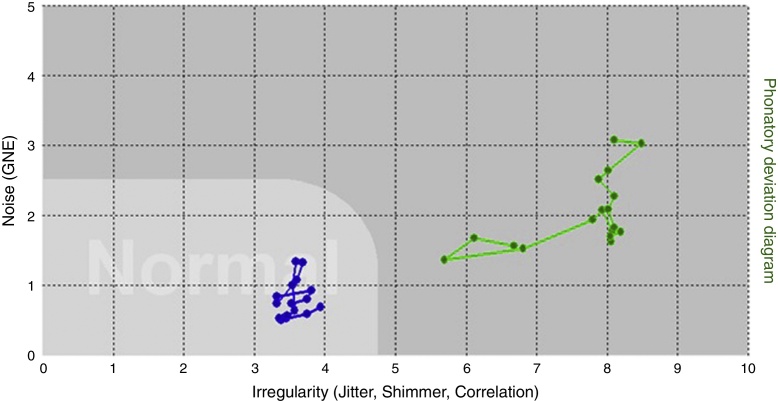
Vocal signals inside (dots in blue color) and outside (dots in green color) the PDD normal area.

Regarding the area, the software itself indicates whether the vocal signal is inside or outside the normal range (Fig. 1).

**Figure 2:**
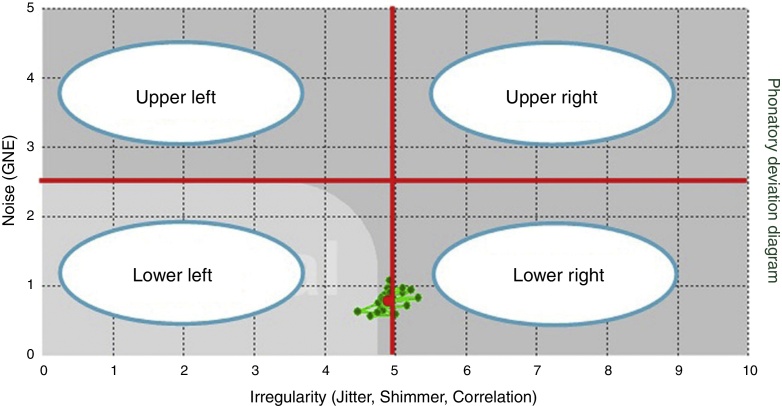
Division of PDD in quadrants.

As for the quadrants, the PDD was divided into four equal quadrants^17^: lower left (1), lower right (2), upper right (3) and upper left (4) (Fig. 2).

**Figure 3:**
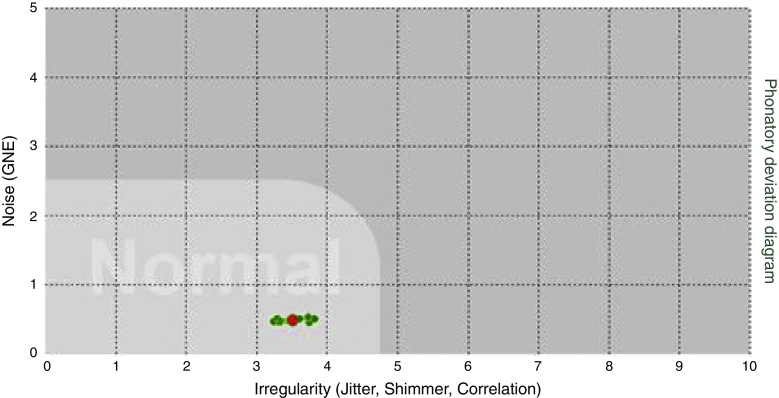
Vocal sample with density concentrated on PDD.

**Figure 4:**
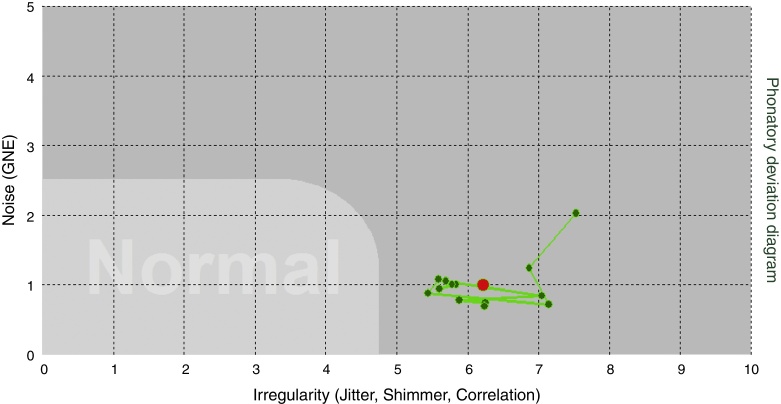
Vocal sample with density amplified in PDD.

Regarding the distribution of the points in relation to density (Figure 3, Figure 4), the points concerning the distribution of the vocal signals were classified as concentrated, when the points were distributed inside a space corresponding to one square, or amplified, when the points were distributed throughout the space corresponding to more than one square of the PDD.

The shape classification was performed using a simple 10-cm ruler on the printed sheet of each PDD generated by the software, corresponding to the image of each analyzed vocal signal, with no previous knowledge of the vocal deviation intensity and the predominant voice type.

The points concerning the distribution of vocal signals were categorized as vertical, when the distance between the points along the abscissa was lower than along the ordinate (*X* < Y); horizontal, when the distance between the points along the abscissa was higher along the ordinate (*X* > *Y*); and circular when the distance between the points along the ordinate and the abscissa was approximately the same (*X* ≅ *Y*).^17^

The perceptual-auditory evaluation session took place in a quiet environment and was performed by a speech therapist/audiologist who was also a voice specialist with more than 10 years of experience in this task.

The evaluator was instructed that voices should be considered normal when they were socially acceptable, naturally produced, without any irregularity, noise, or effort observable during the emission. The evaluator was also instructed that roughness would correspond to the presence of vibratory irregularity and breathiness would be associated with audible air escape during the emission. The evaluator was trained with anchor stimuli, containing normal emissions, and deviated ones at different degrees, as well as predominantly rough and breathy voices. Moreover, the evaluator was instructed about the cutoff values that would be used in this study,^10^ to categorize voices regarding the absence and presence of roughness and breathiness.

For the assessment, the evaluator used a Visual Analogue Scale (VAS), with a metric scale of 0–100 mm, evaluating the intensity of vocal deviation (GD, general degree) and the roughness degree (RD) and breathiness degree (BD). The evaluation closest to 0 represents less vocal deviation, and the closer to 100, the greater the deviations.

For the assessment, each emission of the sustained vowel was presented three times through a speaker, at a comfortable intensity self-reported by the evaluator. At the end of the perceptual assessment session, 10% of the samples (88 signals) were randomly repeated for the evaluator's reliability analysis, using Cohen's Kappa Coefficient. The Kappa value was 0.88, indicating excellent reliability of the evaluator.^35^

In the current literature,^10^, ^36^ distinct cutoff values are found for GD,^36^ RD^10^ and BD,^10^ used to categorize both the presence/absence of vocal deviation, and to classify the degree of the present deviation. Therefore, considering that the aim of this study is to investigate the performance of the PDD in the discrimination of the presence and degree of roughness and breathiness in synthesized voices, it was decided to use the cutoff values established for the classification of roughness and breathiness parameters.^10^

For RD, the following cutoff points are considered^10^: absence of roughness or Grade 0 (0–8.5 mm), mild roughness or Grade 1 (8.6–28.5 mm), moderate roughness or Grade 2 (28.6–59.5) and intense roughness or Grade 3 (≥59.6 mm). In relation to BD, the following cutoff points were recommended: no breathiness or Grade 0 (0–8.5 mm), mild breathiness or Grade 1 (8.6–33.5 mm), moderate breathiness or Grade 2 (33.6–52.0 mm) and intense breathiness or Grade 3 (≥ 52.1 mm).

Thus, a correspondence was made between the VAS used for RD and BD and the numerical scale,^10^ as described below:

Grade 0: RD and BD ≤ 8.4 mm;
Grade 1: 8.5 mm ≤ RD ≤ 28.4 mm and 8.5 ≤ BD ≤ 33.4 mm;
Grade 2: 28.5 mm ≤ RD ≤ 59.4 mm and 33.5 mm ≤ BD ≤ 52.4 mm;
Grade 3: RD ≥ 59.5 mm and BD ≥ 52.5 mm.

The 8.4 mm cutoff was also used to categorize the voices regarding the presence or absence of roughness and breathiness.^10^ Voices with values >8.4 mm in RD and BD were considered as having the presence of roughness and breathiness in vocal emissions, respectively.

We chose not to analyze the tension parameter, since other studies have already shown that such characteristic is not specifically identified in the PDD,^17^, ^29^ in addition to the lack of consensus regarding the inclusion of this parameter in the perceptual-auditory evaluation protocols.^1^, ^10^

The GD evaluation^36^ was not used for signal categorization, but only for the sample characterization in the present study.

Therefore, based on the results of the perceptual-auditory analysis of the RD and BD, the following classification was observed:

As for the presence of roughness: 128 (14.7%) signals without roughness (RD ≤ 8.4 mm) and 743 (85.3%) with roughness (RD ≥ 8.5 mm) (Table 1).

**Table 1:** Distribution of vocal signals regarding the presence and degree of roughness and breathiness.

Variable	*n*	%
*Degree of roughness*		
Normal	128	14.70
Mild to moderate	256	29.40
Moderate	475	54.50
Intense	12	1.40

Total	871	100

*Degree of breathiness*		
Normal	365	41.90
Mild to moderate	187	21.50
Moderate	310	35.60
Intense	9	1.00

Total	871	100
As for the presence of breathiness: 365 (41.9%) signals without breathiness (BD ≤ 8.4 mm) and 506 (58.1%) with breathiness (BD ≥ 8.5 mm) (Table 1).

It is worth mentioning that a categorical analysis of the vocal quality predominant in the emission was not performed, but a same vocal signal could show roughness and breathiness components, since the criterion for the allocation of signals regarding the presence/absence of these components was the result of the independent evaluation of each of them through the VAS (RD and BD) and of the cutoffs established for these parameters (Table 2).

**Table 2:** Comparison of the distribution frequency of the synthesized voices with and without roughness depending on the PDD area, density, quadrant, and shape.

Configuration	Without roughness			With roughness			*p*-Value
	*n*	%	VAS-GD	*n*	%	VAS-GD	
*Area*							<0.001^a^
Inside	82	64.07	20.60 ± 6.66	35	4.71	41.47 ± 23.54	
Outside	46	35.93	26.61 ± 16.05	708	95.28	60.05 ± 0.00	

*Density*							0.060
Concentrated	79	61.71	22.57 ± 17.28	514	69.15	60.92 ± 19.38	
Amplified	49	38.28	23.08 ± 8.66	229	30.82	55.25 ± 0.00	

*Quadrant*							<0.001^a^
Lower left	90	70.31	21.22 ± 8.66	49	6.59	41.15 ± 23.04	
Lower right	38	29.68	26.42 ± 16.05	688	92.59	60.42 ± 0.00	
Upper right	0	0		6	0.80	2.83 ± 0.91	

*Shape*							0.488
Circular	3	2.34	21.00 ± 23.16	27	3.63	68.67 ± 22.14	
Horizontal	125	97.65	22.81 ± 8.66	711	95.69	58.76 ± 0.00	
Vertical	0	0		5	0.67	60.30 ± 24.02	

a Significant values (*p* < 0.05) – Chi-square test and Fisher's exact test. VAS, Visual Analogue Scale; GD, general degree.

### Data analysis

The statistical analysis was descriptive for all the assessed variables and Fisher's exact test and Chi-square test (*x*^2^) were used to compare the analysis of variables related to perceptual-auditory (presence and degree of roughness and breathiness) and acoustic measures (area, density, shape, and quadrant of the PDD). The Kruskal–Wallis test was used to compare the acoustic measurements according to the degree of roughness and breathiness. The level of significance was set at 5% for all analyses. The software used was the Statistical Package for Social Sciences (SPSS, version 21.0).

## Results

Initially, the distribution frequency of the synthesized voices with and without roughness was compared according to the area, density, quadrant, and shape of the PDD (Table 2). A difference was observed between the signals with and without roughness as a function of the PDD area and quadrant (Table 2). The vocal signals with roughness were found to be proportionally outside the area of normal PDD and in the lower right quadrant. There was no statistically significant difference regarding the distribution of the signals with and without roughness as a function of the density and shape of the PDD points.

Subsequently, the distribution of signals with and without breathiness was compared as a function of the PDD parameters. There was a difference in the proportion of these signals regarding the PDD area, density, and quadrant. The breathy voices were predominantly outside the normal range and in the lower right quadrant (Table 3).

**Table 3:** Comparison of the distribution frequency of synthesized voices with and without breathiness as a function of PDD area, density, quadrant, and shape.

Configuration	Without breathiness			With breathiness			*p*-Value
	*n*	%	VAS-GD	*n*	%	VAS-GD	
*Area*							<0.001^a^
Inside	90	24.65	40.90 ± 16.47	27	5.33	44.07 ± 24.86	
Outside	275	75.35	53.35 ± 16.49	479	94.67	58.10 ± 10.32	

*Density*							0.031
Concentrated	236	64.65	8.01 ± 19.09	357	70.55	58.45 ± 10.32	
Amplified	129	35.35	9.13 ± 16.88	149	29.45	54.68 ± 17.42	

*Quadrant*							<0.001^a^
Lower left	103	28.21	5.21 ± 15.04	36	7.12	44.08 ± 25.00	
Lower right	262	71.79	9.66 ± 16.88	464	91.69	58.07 ± 10.32	
Upper right	0	0		6	1.19	80.91 ± 24.83	
Upper left							

*Shape*							0.861
Circular	10	2.74	5.85 ± 25.27	20	3.96	65.55 ± 24.32	
Horizontal	355	97.26	8.84 ± 16.88	481	95.05	56.91 ± 10.32	
Vertical	0	0		5	0.99	64.1 ± 25.87	

a Significant values (*p* < 0.05) – Chi-square test and Fisher's exact test. VAS, Visual Analogue Scale; GD, general degree; PDD, phonatory deviation diagram.

When comparing the distribution frequency of the voices with different degrees of roughness according to the PDD parameters, a difference in the distribution of the signals was observed in relation to all PDD parameters (Table 4). Voices with a higher degree of roughness were proportionally outside the area of normality, in the lower right quadrant and showed concentrated density in relation to voices with lower degrees of roughness. As for the shape, although a difference was found between the proportions of the groups, there was no distribution pattern of the signals with different degrees of roughness in a specific shape, since the signals predominantly showed the horizontal shape in all grades.

**Table 4:** Comparison of the distribution frequency of voices with different degrees of roughness depending on the PDD area, density, quadrant, and shape.

Configuration	Normal (0)			Mild to moderate (1)			Moderate (2)			Intense (3)			*p*-Value
	*n*	%	VAS-GD	*n*	%	VAS-GD	*n*	%	VAS-GD	*n*	%	VAS-GD	
*Area*													<0.001^a^
Inside	82	64.06	20.60 ± 8.66	31	12.10	38.82 ± 23.54	4	0.84	62.00 ± 29.97	0	0		
Outside	46	35.93	25.61 ± 16.65	225	87.89	43.38 ± 0.00	471	99.15	67.21 ± 0.00	12	100	91.37 ± 22.14	

*Density*													
Concentrated	79	61.71	22.57 ± 11.08	153	59.76	43.30 ± 23.54	350	73.68	67.66 ± 0.00	11	91.66	91.50 ± 22.14	<0.001^a^
Amplified	49	29.68	23.08 ± 8.66	103	40.23	42.13 ± 0.00	125	26.31	65.78 ± 19.80	1	8.33	90.00 ± 19.90	

*Quadrant*													
Lower left	90	70.31	21.27 ± 6.66	45	17.57	39.30 ± 23.54	4	0.84	62.00 ± 24.74	0	0		<0.001^a^
Lower right	38	38.28	26.42 ± 16.05	210	82.03	43.53 ± 0.00	466	98.10	67.22 ± 0.00	12	100	91.37 ± 18.04	
Upper right	0	0		1	0.39	47.05 ± 20.93	5	1.05	66.10 ± 20.60	0	0		
Upper left	0	0		0	0		0	0		0	0		

*Shape*													
Circular	3	2.34	21.00 ± 23.16	3	1.17	42.50 ± 18.86	21	4.42	69.40 ± 20.60	3	25.00	90.66 ± 18.04	0.019^a^
Horizontal	125	97.65	22.81 ± 8.66	251	98.04	42.80 ± 0.00	451	94.94	66.98 ± 0.00	9	75.00	97.61 ± 19.90	
Vertical	0	0		2	0.78	46.25 ± 16.63	3	0.63	79. 66 ± 20.08	0	0		

Significant values (*p* < 0.05) – Chi-square test and Fisher's exact test.

VAS, Visual Analogue Scale; GD, general grade; PDD, phonatort deviation diagram.

Regarding the degree of breathiness, there was a difference in the distribution of the signals as a function of the PDD area, density, and quadrant parameters (Table 5). Voices with higher degrees of breathiness were proportionally more often outside the area of normality, showed more concentrated density and were in the lower right quadrant, in relation to the signals with lower degrees of breathiness.

**Table 5:** Comparison of the frequency of voice distribution with different degrees of breathability depending on the PDD area, density, quadrant, and shape.

Configuration	Normal (0)			Mild to moderate (1)			Moderate (2)			Intense (3)			*p*-Value
	*n*	%	VAS-GD	*n*	%	VAS-GD	*n*	%	VAS-GD	*n*	%	VAS-GD	
*Area*													
Inside	89	24.38	4.87 ± 11.55	24	12.83	40.43 ± 22.76	4	1.29	56.37 ± 21.12	0	0		<0.001^a^
Outside	276	75.61	9.55 ± 12.08	163	87.16	42.90 ± 23.22	306	98.70	65.24 ± 7.55	9	100	90.61 ± 22.39	

*Density*													
Concentrated	235	64.38	8.02 ± 13.50	124	66.31	42.50 ± 23.22	225	72.58	65.73 ± 7.55	9	100	90.61 ± 22.39	0.008^a^
Amplified	130	35.61	9.13 ± 12.08	63	33.68	42.75 ± 21.34	85	27.41	63.53 ± 12.96	0	0		

*Quadrant*													
Lower left	102	27.94	5.20 ± 11.55	32	17.11	40.73 ± 22.76	5	1.61	57.90 ± 21.12	0	0		<0.001^a^
Lower right	263	72.05	9.66 ± 12.08	155	82.88	42.96 ± 23.22	300	96.77	65.01 ± 7.55	8	88.88	90.68 ± 22.39	
Upper right	0	0		0	0		5	1.61	79.10 ± 22.20	1	11.11	90.00 ± 21.75	
Upper left	0	0		0	0		0	0		0	0		

*Shape*													
Circular	10	2.73	5.85 ± 22.54	3	1.60	44.16 ± 24.74	17	5.84	69.32 ± 22.20	0	0		0.563
Horizontal	355	97.25	8.48 ± 12.08	184	98.39	42.55 ± 23.22	288	92.90	64.90 ± 7.55	9	100	90.61 ± 22.39	
Vertical	0	0		0	0		5	1.61	64.10 ± 23.61	0	0		

Significant values (*p* < 0.05) – Chi-square test and Fisher's exact test.

VAS, Visual Analogue Scale; GD, general degree; PDD, phonatory deviation diagram.

## Discussion

This study analyzed the performance of the PDD in the discrimination of the presence and degree of roughness and breathiness in synthesized voices. This section was organized with the purpose of clarifying the conclusions of the study according to the raised hypotheses. Didactically, it was decided to analyze the components of roughness and breathiness in subsections.

### PDD performance in the evaluation of the presence and degree of roughness

This study showed that the PDD area and quadrant were able to discriminate between normal signals and signals with roughness. Voices with roughness were predominantly located outside the area of normality and in the lower right quadrant.

Previous studies, carried out with adults’^17^ and children's voices,^29^ corroborate the findings obtained in the present study. Both the lower right quadrant and the PDD area were important to discriminate voices with presence and absence of roughness, showing these two parameters are robust and reliable to evaluate roughness in dysphonic and non-dysphonic voices.

The PDD evaluates signal irregularity in its horizontal position, being associated to the concept of roughness.^24^, ^26^ The greater the irregularity of the vocal signal, the greater its displacement from left to right in the chart. This fact justifies the location of rough voices outside the area of normality and in the lower right quadrant, both in the present study and in previous ones.^17^, ^29^

Additionally, it is emphasized that roughness is one of the universal parameters of the perceptual-auditory evaluation of vocal quality, representing an important characteristic in the identification of the presence of vocal or laryngeal alterations.^37^

Roughness is commonly related to the presence of structural and/or functional alterations in the larynx, such as is seen in cases of edema, vascular dysgenesis, nodular lesions, polyps, or any other component that generates a mass increase in the membranous portion of the vocal folds^38^ and, consequently, irregularities in the vocal fold vibratory pattern. In the acoustic plane, roughness is associated to the jitter and shimmer parameters.^19^

As for the distribution of voices with different degrees of roughness in the PDD, it was verified that vocal signals with a greater roughness component were proportionally outside the area of normality and in the lower right quadrant. Regarding density, signals with moderate and intense deviation predominantly showed concentrated density.

It is noteworthy that 35.93% (*n* = 46) of the synthesized voices without roughness were outside the area of normality, whereas 12.10% (*n* = 31) of the voices with mild-to-moderate degree of roughness were inside the area of normality, that is, the PDD showed a greater confounding factor in the identification of voices without roughness, with a slight deviation in relation to the signals with a higher degree of roughness (moderate and intense).

In traditional models, with the use of algorithms that extract isolated jitter and shimmer measurements, an inverse behavior is observed, as the use of these isolated measures is less reliable in the evaluation of more deviant voices.^15^, ^17^, ^20^, ^24^, ^26^, ^39^, ^40^, ^41^

Regarding density, few studies^17^, ^28^, ^29^ specifically included this parameter for PDD analysis and none of them investigated the distribution of voices with different degrees of roughness as a function of PDD density. Only one of these studies^17^ showed a difference in the distribution of signals with and without vocal deviation regarding density, with the deviated signals characterized as having amplified density.

In other studies where PDD was used,^20^, ^24^, ^26^, ^40^, ^41^, ^42^ the density parameter can be inferred from the distance between the points only on the abscissa axis, being associated with signals with amplified or concentrated density, respectively. All these studies were longitudinal ones and produced a tendency for less dispersion of the points on the post-intervention abscissa axis, although there is great individual variability in this parameter throughout the treatment,^26^ with significant differences being observed only between pre- and post-treatment conditions.

This study showed greater variability in the distribution of the signals without a roughness component or with a mild-to-moderate degree of roughness between the concentrated and amplified densities. This fact confirms the good performance of the PDD in analyzing signals with a wide range of deviation and its reliability in the assessment of the most deviant signals. Additionally, it can be inferred that the PDD density parameter seems to be more robust to qualitatively analyze the patient's evolution regarding the roughness component in vocal emission.

Regarding the shape, although a statistical significance was verified, a distribution pattern of the signals with different degrees of roughness as a function of this PDD parameter was not observed. In all grades, the voices were predominantly horizontal, with differences being observed only between the proportions of the groups. This finding corroborates the literature, as there is a tendency for the signals to show a predominance of the dispersion of the points in the horizontal dimension, regardless of the presence and degree of vocal deviation.^20^, ^24^, ^26^, ^40^, ^41^, ^42^

Even in the original proposal for the classification of vocal signals as a function of the PDD shape, no significant difference was observed between healthy and deviant signals, as well as between different degrees of deviation and between rough, breathy, and tense voices.^17^ Therefore, the shape of the points distributed in the PDD does not seem to be a robust parameter for signal differentiation.

### PDD performance in the evaluation of the presence and degree of breathiness

When comparing the distribution of vocal signals with and without breathiness as a function of the PDD parameters, it was observed that area and quadrant were able to discriminate normal vocal signals from breathy ones. Breathy vocal signals were outside the normal range and were predominantly located in the lower right quadrant.

Breathiness is among the universally accepted parameters for the perceptual-auditory evaluation of vocal quality and for the characterization of a dysphonic voice.^4^, ^8^, ^37^ Thus, the fact that the PDD correctly identifies the breathy signals outside the area of normality reinforces its usefulness in the clinical context of vocal assessment.

However, it was observed that the PDD area and quadrant parameters showed identical behavior, in both rough and breathy voices. The vocal signals with roughness and breathiness were found outside the area of normality and in the lower right quadrant. Therefore, one can discuss the interrelationships of these two parameters in physiological and perceptual terms.

The presence of breathiness is physiologically associated with a higher degree of separation between the vocal processes, lower convexity of the free edge of the vocal folds and the shorter time of the closed phase of the glottic cycles^43^ In turn, vocal folds that are further away from the midline tend to vibrate with greater irregularity and less amplitude of the mucosal wave,^44^ which, consequently, generates the roughness component in the emission.^37^

Therefore, considering that the signals with roughness and breathiness showed, in general, moderate deviation, with GD of 62.19 ± 14.80 and 65.28 ± 14.75 points in the VAS,^36^ respectively, one understands the similar distribution of signals with roughness and breathiness in the PDD area and quadrant.

Although the synthesizer used to generate the signals in this study allows the creation of voices with isolated components of roughness (disturbance) and breathiness (additive noise), this separation was not used in the present study. We suggest further investigations with separation of the exclusively rough and breathy signals to assess the performance of the PDD in this classification.

In other studies,^17^, ^29^ the breathy voices were located outside the area of normality, but were distributed between the lower right and upper right quadrants. Some methodological issues need to be highlighted to evidence the similar distribution of the rough and breathy voices in the lower right quadrant in this study.

The two aforementioned studies^17^, ^29^ used as a criterion to classify the voices as rough, breathy, or tense, a forced choice task, in which the evaluator, if he/she considered the emission deviant, should determine the predominant vocal quality. This type of evaluation task allows only one possibility of choice for each emission and not necessarily a classification regarding the presence/absence of each deviated parameter in the emission.

In turn, the present study evaluated the degree of roughness and breathiness present in the emission through a VAS. Based on the cut-off values, the presence/absence of such components was established, with the possibility that the same signal would concomitantly show the presence of one or more of them, which is close to the usual conditions of deviant vocal production.

Another finding of this research is the high percentage of voices without breathiness (75.40%; *n* = 276) classified outside the normal range of the PDD. In a qualitative data analysis, it can be observed that the GD of deviation of these signals is 53.35 ± 16.49. Therefore, although these signals did not show auditory-perceived breathiness, they were probably evaluated as deviated in the VAS due to the presence of roughness in the emission.

When comparing the results regarding the proportion of voices with presence/absence of roughness and presence/absence of breathiness identified inside and outside the PDD normality area, it is observed that there is a greater identification of voices without roughness within the area of normality (64.07%, *n* = 82) and a greater identification of voices without the breathiness component outside the normality area (75.35%; *n* = 275).

Qualitatively, a difference of more than 20 points was found regarding the VAS GD between voices without roughness and without breathiness, with higher GD values in the latter group. This difference in itself would justify the results regarding the higher proportion of signals without the breathiness component identified outside the normal range.

These findings reinforce that, even in conditions where the perceptual-auditory evaluation criteria used to classify the signals were not intended to maximize the differences between them, but to evaluate them over a continuum, the PDD was also efficient for vocal evaluation, mainly in relation to the most deviant signals.

It is suggested that other studies be carried out using the same methodology and criteria of perceptual-auditory evaluation used in this study, adding to them the criterion that the signals selected for investigation have only one of the components deviated from the cutoff values of the VAS.

Regarding the degree of breathiness, there was a difference in the distribution of the signals as a function of the PDD area, density, and quadrants. It was observed that the higher the degree of breathiness, the greater the proportion of signals located outside the area of normality, in the lower right quadrant and with concentrated density. Therefore, it is verified that the greater the breathiness component in the vocal signal, the greater the capacity of the PDD to correctly identify the presence of the deviation.

As previously mentioned, such finding regarding the classification of signals with higher degree of deviation constitutes one of the greatest advantages of the PDD, as it fills an existing gap^15^ regarding the use and reliability of traditional measures of disturbance and noise in the evaluation of voices with moderate and intense deviations.

Once again, a similar distribution of the voices with different degrees of roughness and breathiness was observed as a function of the area, quadrant, and density of the PDD. The only difference between the voices with different degrees of roughness and breathiness is the distribution of the signals with Grade 2, in which there was a higher level of correct identification of the group of voices without roughness within the PDD normality area. This fact has already been discussed in this section.

The vertical axis of the PDD evaluates the presence of additive noise in the vocal signal, compatible with the presence of the breathiness component.^26^ Therefore, it was expected that the higher the breathiness component in the emission, the greater the proportion of signals toward the upper left quadrant.

In the study^17^ with voices of dysphonic adults, it was observed that breathy voices, although they were predominantly distributed in the upper left quadrant (52.6%; *n* = 30); 19.3% (*n* = 11) were also situated in the lower right quadrant. With the pediatric population,^29^ breathy voices were distributed in the lower right (35%, *n* = 7), lower left (30%, *n* = 6), upper right (30%, *n* = 6) and upper left (5%, *n* = 1) quadrants.

In studies^26^, ^41^ with patients presenting with unilateral vocal fold paralysis^26^ and individuals with bilateral vocal fold paralysis,^26^, ^41^ it was found that only the second group, whose patients showed intense breathiness, had their voices located in the upper right quadrant. In turn, individuals with unilateral paralysis had their voices distributed between the lower left and lower right quadrants.^26^

In general, in high lesions of the vagus nerve, the vocal folds are more distant from the midline and the vocal emission does not originate from the glottic vibration mechanism, but comes primarily from the turbulent transglottic airflow and its propagation in the vocal tract,^45^, ^46^ which would justify the presence of these signals in the upper right quadrant.^26^

In the present study, only nine signals were classified as having severe breathiness deviation, and of these, only one (11.11%) was in the upper right quadrant. In this way, two points can be highlighted: first, the sample size, since a different result could have been observed in this distribution with a larger sample of breathy voices with intense deviations; second, as already emphasized in the discussion, there is an overlap of the type of vocal deviation in the assessed signals, since the presence of only one type of deviation in each emission was not used as eligibility criterion.

## Conclusion

The PDD area and quadrant can discriminate the presence and absence of roughness, as well as the presence and absence of breathiness in synthesized voices. Signals with higher degree of roughness and breathiness are proportionally outside the area of normality, in the lower right quadrant and with concentrated density.

## Conflicts of interest

The authors declare no conflicts of interest.
